# Comparison Between Ultrasound and High-Pressure Homogenization for Encapsulation of β-Carotene in CNF-Stabilized Pickering Emulsions

**DOI:** 10.3390/polym18010126

**Published:** 2025-12-31

**Authors:** Adila Abdirym, Xue Wu, Bin Liu

**Affiliations:** School of Computer and Artificial Intelligence, Beijing Technology and Business University, Beijing 100048, China; 18167413740@163.com

**Keywords:** β-carotene, Pickering emulsion, high-pressure homogenization (HPH), ultrasounds (US), encapsulation

## Abstract

This work investigated the stabilization mechanisms and β-carotene encapsulation characteristics of cellulose nanofibrils (CNFs) stabilized Pickering emulsions (PE) prepared by different emulsification processes. For 48 days of storage, ultrasound-prepared Pickering emulsions (US-PE) stabilized by at least 2.0 wt.% CNFs have obvious cream stabilization, and high-pressure homogenization-prepared Pickering emulsions (HPH-PE) stabilized by over 1.6 wt.% CNFs have excellent cream stabilization. The stabilization of HPH-PE, which was superior to that of US-PE, mainly relied on the steric stabilization of CNFs’ space networks. Although the encapsulation efficiency of β-carotene in US-PE was higher than that in HPH-PE when the CNF concentration was over 1.2 wt.%, the retention rate of β-carotene in US-PE was obviously lower than that in HPH-PE. So, the internal space structure of CNF-stabilized HPH-PE was conducive to stabilizing the emulsion and protecting the bioactive molecule.

## 1. Introduction

Emulsions stabilized by colloid particles, which are regarded as Pickering emulsions (PE), have recently appealed to researchers [1]. Because of the advantages in controlled release and delivery, high stability against coalescence, and bioavailability of lipophilic substances [2,3,4,5], Pickering emulsions have seen extensive use in commercially adapted food matrices and carrier systems for bioactive compound entrapment, preservation, and controlled liberation [3,6,7]. As a carotenoid, β-carotene serves as a potent provitamin A precursor [8]. However, given that the human body is unable to synthesize β-carotene, humans can only receive β-carotene from food or health products [9]. The key challenges in utilizing β-carotene as a versatile food constituent are its suboptimal solubility in water, limited stability, and low bioavailability [10]. Therefore, to address the challenges, encapsulation strategies leveraging lipid-based systems have been engineered, restricting β-carotene in the hydrophobic groups of Pickering emulsions.

There are reports revealing that β-carotene was sealed efficiently in Pickering emulsions, which were stabilized by aggregated spherical particles such as the wheat gluten nanoparticle (WGN) [11], non-spherical polydisperse particles such as chitosan-phytic acid-cyclodextrin (CS-PA-CD) nanoparticles [12], complex nanoparticles such as zein-propylene glycol alginate-tea saponin (zein-PGA-TS) [13]. Moreover, the chemical degradation rate of β-carotene was reduced in Pickering emulsions, while its bioavailability and nutritional delivery stability were improved [14,15]. Different from spherical or coil-like composite nanoparticles used as emulsion stabilizer, rod-like nanoparticles, cellulose nanofibrils (CNFs) have a higher aspect ratio and can form a network with a three-dimensional structure or further anchoring matrixes, directing the distribution of oil droplets [16,17,18]. Furthermore, CNFs possess biocompatibility, low toxicity, renewability, and both hydrophilic and hydrophobic groups [19]. Thus, CNFs are gradually becoming preferred as interfacial reservoirs of Pickering emulsions for encapsulating bioactive components [20,21,22].

High-speed homogenization (HSH), ultrasound treatment (US), and high-pressure homogenization (HPH) are the usual emulsification methods. HSH served as a regular high-energy method to prepare Pickering emulsion for encapsulating β-carotene [11,12,15]. Yet the size of oil droplets created by HSH was unchanged tens of microns [23] or increasing from single-digit microns to a dozen microns [22] for 30 days of storage. The oil droplet size was smaller, and the Pickering emulsion became more stable by using US [24] or HPH [25]. Emulsions in which dialdehyde cellulose (DAC) was used as an interfacial stabilizer to deliver lipophilic compounds were prepared by US, and the oil droplet size was a dozen microns or a few hundred nanometers [7]. HPH was also employed as a more efficient method for encapsulating β-carotene in Pickering emulsion stabilized by CNFs about 70 MPa [14] or about 100 MPa [13].

However, the difference between US and HPH for encapsulating and delivering β-carotene wrapped in Pickering emulsion stabilized by CNFs remained unclear. The purpose of this work is to make comparisons between US and HPH as different high-energy emulsifying methods in terms of droplet stability, bioactive components protection, and delivery for β-carotene in Pickering emulsion stabilized by CNFs.

## 2. Materials and Methods

### 2.1. Materials

Cellulose (25 μm, (C_6_H_10_O_5_)_n_, molecular weight 162n) powder was supplied from Aladdin Co., LLC. (Shanghai, China). Soybean oil (average molecular weight 859) was provided by Macklin Biochemical Co., Ltd. (Shanghai, China). β-carotene and n-hexane were obtained from Mreda Technology Co., Ltd. (Shanghai, China). Alcohol was obtained from Sinopharm Chemical Reagent Co., Ltd. (Shanghai, China). All reagents were of chemical grade and used directly without further processing.

### 2.2. Methods

#### 2.2.1. Preparation for Cellulose Nanofibrils (CNFs) Suspension

CNFs suspensions were prepared following relevant articles [26,27]. Firstly, Cellulose powder was dispersed in water at given concentrations of 0.4, 0.8, 1.2, 1.6, and 2.0 wt.%. The cellulose fibers were dispersed in by a magnetic stirrer (Model 601, Shanghai Sanxin Instrument Factory, Shanghai, China) at 500 rpm and then stored at room temperature for 24 h to guarantee complete cellulose swelling. Subsequently, cellulose suspensions were subjected to nanofibrillation using high high-pressure homogenizer (NCJJ-0.005/150, Langfang General Machinery Manufacturing Co., Ltd., Hebei, China) at 110 MPa for three times. Finally, the obtained 0.4, 0.8, 1.2, 1.6, and 2.0 wt.% CNFs suspensions were stocked at 4 °C for further use.

#### 2.2.2. Fabrication of β-Carotene-Loaded CNF-stabilized Pickering Emulsions

Briefly, β-carotene was scattered in soybean oil at 0.05 wt.% via 30 min magnetic stirring in an 80 °C-water bath to ensure that β-carotene was completely solubilized. Next, β-carotene-loaded soybean oil was combined with 0.4, 0.8, 1.2, 1.6, and 2.0 wt.% CNF suspension at 1:9 (*V*/*V*). Subsequently, the pre-emulsions were fabricated via mechanical shearing (13,500 rpm, 2 min) by applying a high-shear stirrer (IKA T18 digital ULTRA TURRAX^®^, Staufen, Germany). The resulting β-carotene-loaded coarse emulsion samples were labeled CE0.4, CE0.8, CE1.2, CE1.6, and CE2.0 in accordance with the original 0.4,0.8,1.2,1.6,2.0 wt.% CNFs suspension. Thereafter, two approaches were used for emulsion processing.

High-pressure homogenization (HPH): Partial β-carotene-loaded coarse emulsion samples labeled CE0.4, CE0.8, CE1.2, CE1.6, and CE2.0 were homogenized once at 80 MPa by using a high-pressure homogenizer (NCJJ-0.005/150, Langfang General Machinery Manufacturing Co., Ltd.). A cool water cycle system was used for maintaining the emulsion temperature < 25 °C. The HPH-prepared β-carotene-loaded CNF-stabilized Pickering emulsion samples were labeled “HPH-PE.” The available HPH-PE samples derived from CE0.4, CE0.8, CE1.2, CE1.6, and CE2.0 were HPH-PE 0.4, HPH-PE0.8, HPH-PE1.2, HPH-PE1.6, and HPH-PE2.0.

Ultrasound treatment (US): The other β-carotene-loaded coarse emulsion samples labeled CE0.4, CE0.8, CE1.2, CE1.6, and CE2.0 were treated with an ultrasound probe at 20 kHz and 180 W for 16 min and 9 s to use the same energy density as the HPH process. A cool water cycle system was used for maintaining the emulsion temperature < 25 °C. The US prepared β-carotene-loaded CNF-stabilized Pickering emulsion samples were labeled “US-PE.” The available samples derived from CE0.4, CE0.8, CE1.2, CE1.6, and CE2.0 were US-PE 0.4, US-PE0.8, US-PE1.2, US-PE1.6, and US-PE2.0.

### 2.3. Characterization

#### 2.3.1. Particle Size and ζ-Potential Measurements

The oil globule size of HPH-PE and US-PE samples was measured using a laser scattering size analyzer (Malvern Instruments Mastersizer 2000, Worcestershire, UK). Refractive indices of the emulsions were 1.38 for the oil phase and 1.33 for the aqueous phase. Particle size data is reported as the volume-weighted mean diameter D [4,3] and the area-weighted mean diameter D [3,2]. The span values were applied to assess the degree of polydispersity in the distribution of droplet size in the emulsions. All measurements were performed at least three times.

The oil droplet surface charge of HPH-PE and US-PE samples was assessed by electrophoretic mobility analysis (Malvern Instruments Zetasizer PRO, Worcestershire, UK) in an electric field. In order to mitigate multiple scattering effects, deionized water was used to dilute emulsions to reach 0.1 wt.% oil droplets. After 60 s equilibration, ≥10 sequential readings were recorded per sample. The aforementioned tests were conducted in triplicate.

Particle size and ζ-potential measurements were conducted immediately after the samples were prepared.

#### 2.3.2. Optical Microscopy

The oil droplets’ morphology of HPH-PE and US-PE samples was determined with an optical microscope (BK1201, Chongqing Optical Instrument Factory, Chongqing, China). The prepared samples were diluted 100 times, and a small droplet was deposited between a microscopy slide and a coverslip to prepare specimens for microscopy observation. The morphology of the oil droplets was observed by a Swift fit digital camera (SC1603-CK, Motic (Xiamen) Network Technology Co., Ltd., Xiamen, China). Optical microscopy was performed within 2 h after the samples were prepared.

#### 2.3.3. Creaming Stability

The creaming index (CI) of HPH-PE and US-PE samples is usually used to indicate the creaming stability. It serves as an indirect index of droplet aggregation dynamics in emulsion systems, where higher values are in line with greater aggregation. CI values of diverse emulsions were established instantly after the emulsification processes. Each emulsion sample was placed into a 25 mL glass bottle and stored at room temperature (about 22 °C). The serum layer height (Hs) at the tube bottom and total emulsion height (Ht) were documented after quiescent storage. The test was repeated in three replicates. CI was calculated according to Equation (1) using the method described in earlier investigations [28]:

(1) $$CI\left(\%\right)=\frac{Hs}{Ht}\times 100\%$$

#### 2.3.4. Rheological Characterization

All rheological properties of HPH-PE and US-PE samples were described by applying a Discovery Hybrid Rheometer (TA Instruments Discovery HR-2, New Castle, DE, USA), which was furnished with a parallel-plate configuration (diameter 40 mm) and a solvent trap. When shear rate (γ, 1/s) ranged from 0.01 to 1000 1/s, the apparent viscosity (ηa, Pa s) of each sample was measured three times to indicate steady shear properties of the emulsions.

#### 2.3.5. Encapsulation Efficiency (EE) of β-Carotene

The encapsulated β-carotene in CNF-stabilized Pickering emulsions was recovered by a solvent extraction technique [11]. 3 mL n-hexane and 2 mL ethanol were added to each sample (1 mL), and the mixtures were oscillated for 30 s. Then samples were equilibrated for 5 min to reach complete phase separation. The hexane phase (the yellow supernatant) was transferred to a centrifuge tube. The above extraction process using 3 mL n-hexane was duplicated twice. Finally, the ethanol phase showed no coloration. The extracts were volumetrically standardized to 10 mL with n-hexane. Then, the insoluble components were removed by centrifugation (5 min, 4000 g). The absorbance of the extracts was surveyed at 450 nm using a spectrophotometer (Shmadzu UVmini-1240, Kyoto, Japan). Each sample was analyzed three times. The β-carotene concentration was defined against a standard curve, which was established under the same conditions in advance, with encapsulation efficiency (EE) calculated using Equation (2) [29]. EE was calculated as the β-carotene concentration percentage: C_initial_/C_total_ (%), where C_initial_ represents the initial β-carotene concentration present in the emulsified sample before storage, and C_total_ denotes the total β-carotene concentration in the non-emulsified sample.

(2) $$Encapsulation\;efficiency\;(EE)=\frac{C_{initial}}{C_{total}}\times 100\%$$

#### 2.3.6. Retention Rate (RR) of β-Carotene During Storage

The US-PE and HPH-PE samples, which were reposited in the laboratory at room temperature, were exposed to natural light indoors. β-carotene degradation kinetics were characterized by periodic concentration measurements throughout storage. β-carotene concentration was calculated by the way of Section 2.3.5, and retention rate (RR) was computed according to Equation (3), the β-carotene concentration percentage: C_storage_/C_initial_ (%). C_storage_ is β-carotene concentration after defined storage intervals, and C_initial_ is the initial β-carotene concentration present in the emulsified sample before storage.

(3) $$Retention\;rate\;(RR)=\frac{C_{storage}}{C_{initial}}\times 100\%$$

#### 2.3.7. Statistical Analysis

Experimental trials were performed in triplicate, and the resulting data were presented as mean values ± standard deviations. Statistical analysis was executed employing Origin 2022 (Stat-Ease Company, Minneapolis, Minnesota, USA), with significance determined at *p* < 0.05.

## 3. Results

### 3.1. Formation of c CNF-stabilized Pickering Emulsions

**Figure 1:**
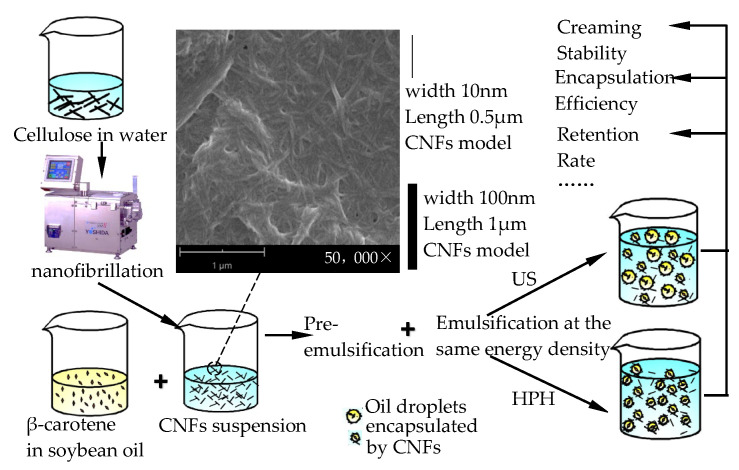
The process flow diagram used for obtaining β-carotene-loaded CNFs-stabilized Pickering emulsions.

The preparation and formation mechanism of β-carotene-loaded CNFs-stabilized Pickering emulsions are demonstrated in Figure 1. According to Section 2.2.1, the prepared CNFs in suspension had a high aspect ratio (10–100 nm wide, 0.5–2 μm in length). It was indicated that the sizes were fit for the formation of a nanofibril network in the emulsions, which further promoted the emulsion stability [20]. In accordance with previous studies, oil droplet deformation and breakage occurred in coarse emulsion by a high-shear mixer through mechanical shearing. Prior studies have employed prepared pre-emulsions for ultrasound emulsification [7,29] or high-pressure homogenization [13,14] to encapsulate bioactive molecules.

The energy density provided by the HPH and US process was set to be equal to compare the characteristics and properties of β-carotene-loaded CNF-stabilized Pickering emulsions. The calculation of the energy density was determined as described by Calligaris et al. [30] and Bot et al. [31]. Yet, high pressure in the experimental NCJJ-0.005/150 homogenizer was discontinuous and periodic, and thus, HPH energy density was calculated with Equation (4). The operating pressure was about 80 MPa under the condition of 0.1 m/s piston speed, and the pump pressure acting area was 7.85 × 10^−5^ m^2^ (the diameter of the pump piston is 10 mm). The time for which high pressure was kept through the pump piston added up to about 250 s for processing a total of 200 mL coarse emulsion. The electric efficiency of HPH equipment is about 90%. Thus, HPH energy density was 872 J/cm^3^ according to Equation (4). In the US process experiments, as the control, the applied power is 180 W for 16 min and 9 s. Thus, the US energy density for the 200 mL emulsion weight was also 872 J/cm^3^ according to Equation (5). The energy density of the HPH and US processes is coincident.

(4) $$\begin{array}{l} HPH\;energy\;density\left(\frac{J}{{cm}^{3}}\right)\\ =\frac{pressure\left(Pa\right)\times acting\;area\left(m^{2}\right)\times pump\;speed(m/s)\times Time(s)}{electric\;efficiency\;of\;equipment\left(\%\right)\times emulsion\;volume({cm}^{3})}\times 100\% \end{array}$$

(5) $$US\;energy\;density\left(\frac{J}{{cm}^{3}}\right)=\frac{Nominal\;applied\;power(W)\times Time(s)}{Volume\;of\;US\;processed\;emusion({cm}^{3})}\times 100\%$$

### 3.2. Characterization of β-Carotene-Loaded CNF-stabilized Pickering Emulsions

**Figure 2:**
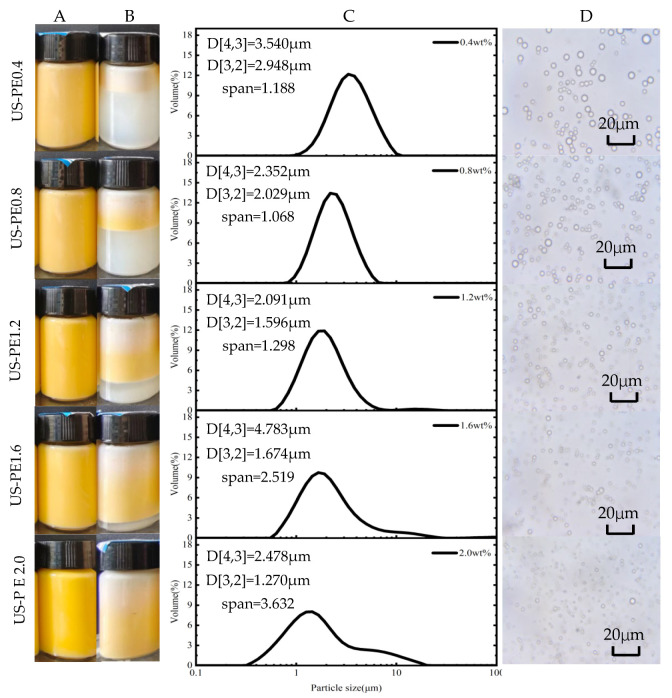
β-carotene-loaded CNF-stabilized Pickering emulsions made using US emulsification processes: the appearance of the samples (**A**) finished and (**B**) 48d storage; (**C**) particle size distributions, d[3,2] and d[4,3], and (**D**) light microscopy images of the finished samples.

According to the appearance of β-carotene-loaded CNF-stabilized Pickering emulsion obtained by the US process, with the increased CNFs concentration up to 2.0 wt.%, the stratified phenomenon of the emulsion declined until it finally did not occur (Figure 2A,B). It was confirmed that the stability of the emulsion made using the US process improved with the increased CNF concentration.

The impact of CNF concentrations on the particle size distributions and the microstructure of β-carotene-loaded CNF-stabilized Pickering emulsions obtained by the US process is presented in Figure 2C,D. The particle size distribution curve moved to the left, and span values increased with the increased CNF concentration (Figure 2C). Generally, the overall interfacial area of the emulsion, which was applied to evaluate emulsification performance or efficiency, can be determined using the area-weighted mean diameter d[3,2]. And the smaller the d[3,2] of the emulsion, the better the emulsification performance and the higher the emulsification efficiency. The area-weighted mean diameter d[3,2] decreased significantly to 1.270 ± 0.030 μm, and the span increased to 3.632 ± 0.318 μm in US-PE2.0 samples. The phenomena indicated that emulsion droplets were reduced and but the droplets uniformly decreased with the increased CNFs concentration. Analogous literature also suggested that, with the ascent of nanoparticle concentration, the oil droplet size of β-carotene and curcumin co-embedded emulsion shows a descending trend [12,13].

**Figure 3:**
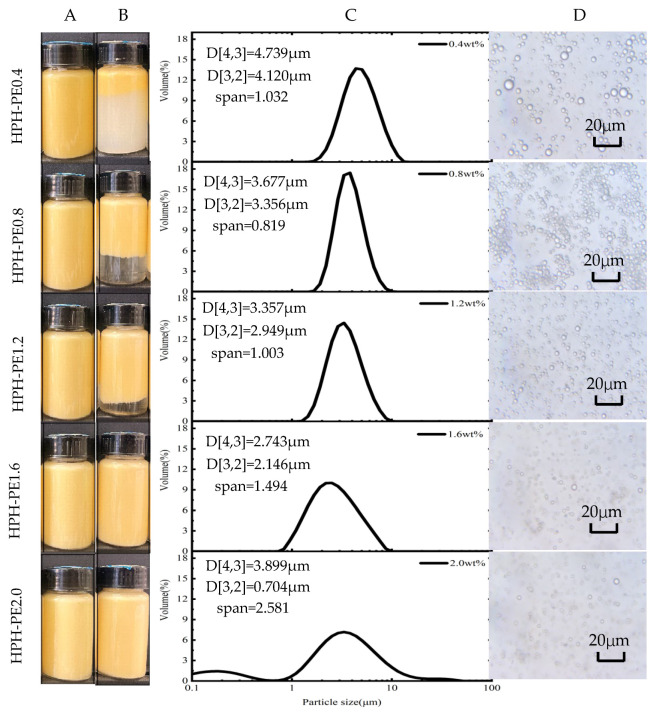
β-carotene-loaded CNF-stabilized Pickering emulsions made using HPH emulsification processes: the appearance of the samples (**A**) finished and (**B**) 48d storage; (**C**) particle size distributions, d[3,2] and d[4,3], and (**D**) light microscopy images of the finished samples.

When processed by HPH at the same energy density as US, β-carotene-loaded Pickering emulsions, which were stabilized by over 1.6 wt.% CNFs suspension for 48 days storage, were very stable and the layering phenomena did not occur (Figure 3A,B). The photolysis of β-carotene on the upper part of the emulsions obtained by the HPH process was negligible or even absent relative to that by the US process (Figure 2B and Figure 3B).

The area-weighted mean diameter d[3,2] of the Pickering emulsion, which was stabilized by 2.0 wt.% CNFs suspension and obtained by the HPH process decreased significantly to 0.704 ± 0.005 μm, and the emulsion droplets were much smaller than those of the other samples (Figure 3C,D). This suggests an enhanced stability of this emulsion because a smaller droplet size decelerates destabilization processes, such as creaming and flocculation. According to Stokes’ law, the droplet velocity is proportional to the square of droplet radius; therefore, the emulsion stability, which relates to phase separation, can be elevated by the reduction in droplet size [32]. In summary, 1.6 wt.% CNFs suspension was enough for stabilizing the Pickering emulsion made using the HPH process.

Steric stabilization and electrostatic stabilization are two main mechanisms for stabilizing Pickering emulsions. In summary, amid the process, one of the mechanisms may predominate over another. The characteristics of steric stabilization are that macromolecules accumulate on the surface of the oil particle. The characteristics of electrostatic stabilization are that the surface charge density of the Pickering emulsion differs. It is known as electrosteric stabilization when steric stabilization and electrostatic stabilization arise simultaneously.

**Figure 4:**
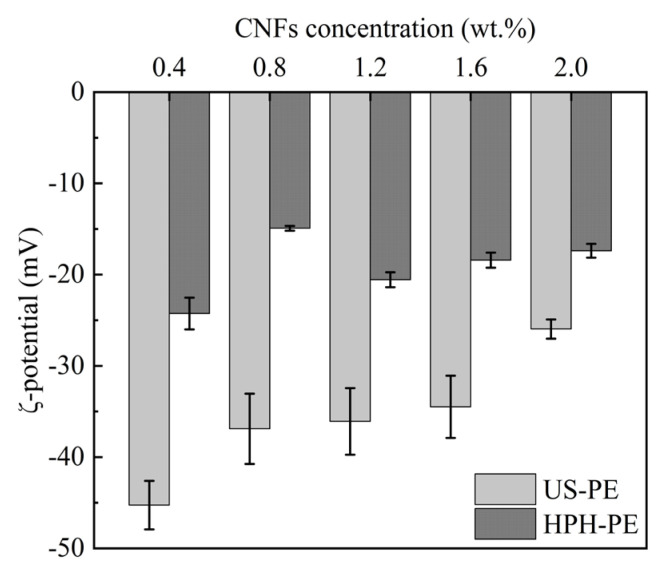
ξ-potential of β-carotene-loaded CNF-stabilized Pickering emulsions.

The ξ-potential is used to measure the extent of electrostatic stabilization. Figure 4 shows the changes in the ξ-potential when the pH of the β-carotene-loaded CNF-stabilized Pickering emulsions was around 6.0. The resulting emulsion droplets, which were obtained by the US process, had obviously higher negative surface charge than those which were obtained by the HPH process. Figure 4 indicates that the electrostatic stabilization of US-PE samples was more significant than that of HPH-PE samples at the same energy density. The altered surface charge density of these CNFs was attributed to the differences in droplet interfacial coverage, the decomposition of liquid molecules, or the fracture of the CNFs’ molecular chains provoked by the strong shock from ultrasonic cavitation [33,34]. It should also be noted that the ξ-potential in US-PE samples declined when the CNF concentration increased from 0.4 to 2.0 wt.% (Figure 4). This decline may be attributed to the increased coverage of CNF’s surfaces by excess cellulose chain molecules, which generate a shielding effect or even restrict the movement of charges, thereby reducing the absolute value of the negative charge.

**Figure 5:**
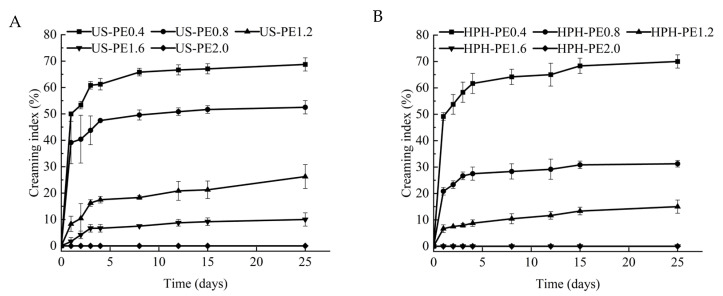
Creaming index of β-carotene-loaded CNFs Pickering emulsions made using (**A**) US; (**B**) HPH.

The difference between US and HPH emulsification for the cream index (CI) of Pickering emulsion is shown in Figure 5A,B. At the same CNF concentration, the degree of phase separation of the Pickering emulsion by HPH was lower than that by US, and the Pickering emulsion system was more stable. These findings imply that the electrostatic mechanism does not dominate in stabilized Pickering emulsions, as the ξ-potential of CNF-stabilized Pickering emulsions made by HPH was lower (Figure 4), but the CI value of these emulsions was higher (Figure 5). There should be much better steric stabilization caused by CNF–encapsulating-oil networks in Pickering emulsion by HPH. It can be inferred that electrosteric stabilization occurred, but the steric contribution showed ascendancy due to the low surface charge in the CNF-stabilized Pickering emulsion made by HPH. Those results were in accordance with the reports that the CI value of CNF-stabilized Pickering emulsion prepared by HPH retained 0% [35], and the CI values of CNF-stabilized Pickering emulsion prepared by US changed from 4.65 ± 0.3% to 13.8% ± 0.1% after 60 days of storage [29].

It follows that steric stabilization should be more effective than electrostatic stabilization for Pickering emulsions. Compared with the US, the HPH process can provide better steric stabilization for Pickering emulsion, and so the HPH-prepared Pickering emulsion is more stable. Although steric stabilization was formed to some extent by the US process, the increase in energy density may prominently improve electrostatic stabilization, which did not work as well for emulsion stabilization as steric stabilization.

### 3.3. Rheological Properties

**Figure 6:**
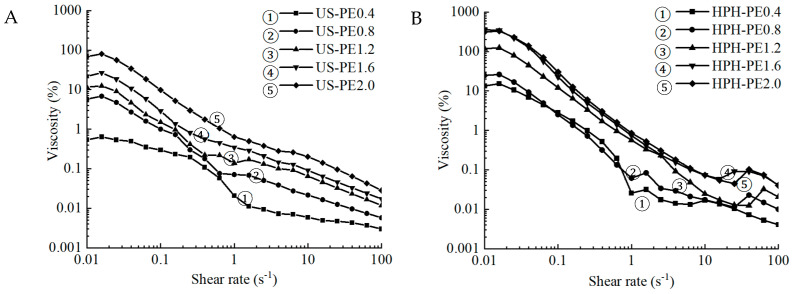
Apparent viscosity of Pickering emulsions made using (**A**) US; (**B**) HPH.

The relationship between the apparent viscosity and the shear rate of the emulsions obtained using US and HPH processes is presented in Figure 6. The HPH-PE2.0 samples showed the highest viscosity among all the emulsions (Figure 6). As processed according to Section 2.2.1, the nanofibrils (CNFs) suspension formed 3D networks [35] and exhibited a gel-like behavior arising from the fibril entanglement. Following Section 2.2.2, CNFs enabled the entrapment of lipid droplets into cellulose aggregates during the incorporation into the emulsion, which resulted in the significantly higher viscosity of CNFs Pickering emulsions when the CNF concentration was elevated from 0.4 to 2.0 wt.%. This is due to the larger amount of nanofibrils at higher CNF concentration, forming more interactions between the nanofibrils. Thus, greater entanglement and thereby greater flow resistance were presented. This was in line with studies and also confirmed the sufficient effects of CNFs on particle size and encapsulation efficiency brought by its microfibril 3D network [36,37,38].

### 3.4. Encapsulation Efficiency of β-Carotene

**Figure 7:**
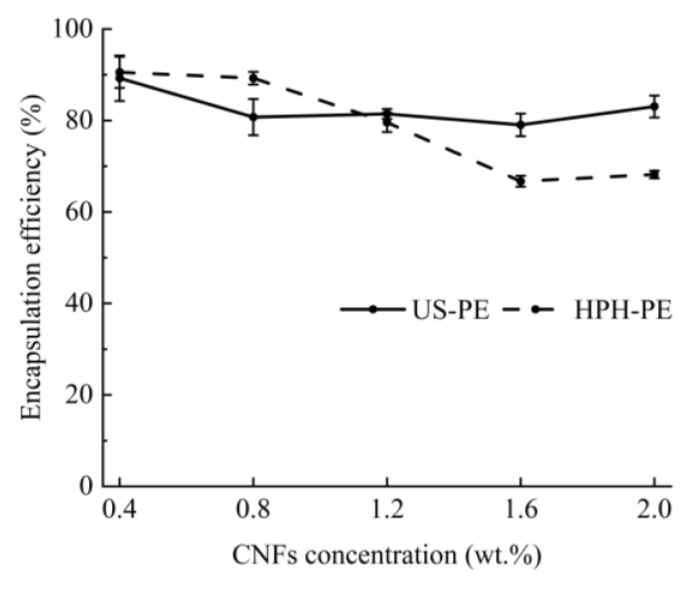
Encapsulation efficiency of β-carotenes.

According to Figure 7, the encapsulation efficiency fell within the range of 79–83% in US-PE samples stabilized by above 1.2 wt.% CNF suspensions in this study (*p* > 0.05), which was also observed by Wei et al. [13]. It showed that the EE of β-carotenes in the US process would not be affected by the raised CNF concentration. These β-carotene-loaded US-PE samples stabilized by over 1.2 wt.% CNF suspensions showed a little higher β-carotene encapsulation efficiency than those emulsions made using the HPH process (*p* < 0.05), highlighting successful encapsulation of β-carotenes by the US process. The encapsulation efficiency decreased from 79.58 ± 2.11% to 66.71 ± 1.21% in HPH processes along with the increased CNF concentration from 1.2 to 1.6 wt.% (*p* < 0.05). This trend was different from that in the references [35,39]. The results should attribute that the added CNF amounts affected the HPH process. Because the CNFs’ entanglement and agglomeration in the HPH process will significantly rise with the raised CNF concentration, the thermal dissipation will lead to the loss of encapsulated β-carotene.

### 3.5. Storage Stability

**Figure 8:**
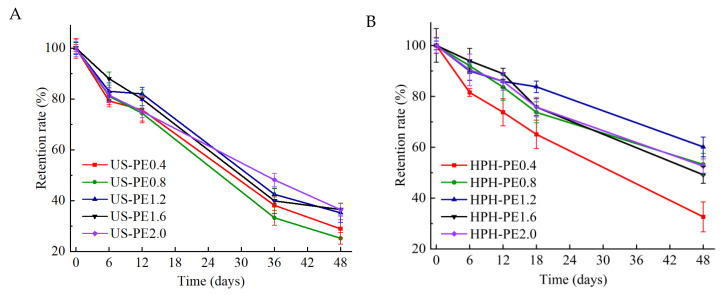
Retention rate of β-carotenes in CNF-stabilized Pickering emulsions made by (**A**) US; (**B**) HPH.

As shown in Figure 8A,B, the retention rate (RR) of β-carotene in US-PE samples decreased more rapidly in the storage period relative to that of HPH-PE samples. After 48 days of storage at room temperature, RR of β-carotene in US-PE1.6, US-PE2.0 samples fell to 36.49 ± 2.49%, 36.41 ± 2.50%; and that in corresponding HPH-PE1.6, HPH-PE2.0 samples fell to 49.16 ± 3.34%, 52.60 ± 2.81%. The results demonstrated that the HPH process could effectively protect bioactive components compared to the US. It could be attributed to the difference in the impact force between the US and HPH. At the same energy density, the impact force of the HPH process continuously acted on the emulsion droplets, and that of the US process relies on the location and degree of cavitation effect. Thus, more interfacial areas and more uniform droplets could be formed in the HPH process, which was beneficial to emulsion stabilization. The above factors jointly contributed to effective component protection. At the same storage time, RR in both US-PE and HPH-PE samples scaled up with the increased CNFs concentration, indicating that the storage stability of both US-PE samples and HPH-PE samples was relative to the CNFs amounts. The results confirmed that adequate CNF concentrations form protective interfacial coatings on oil droplets, enhancing β-carotene core stabilization.

The stable space networks of CNFs should be the foundation of the relatively higher β-carotene retention rate in the HPH emulsions, because the substantial interface formed by the CNF networks could insulate oxides. The prior report [40] also revealed that the extensive preservation of β-carotene in stabilized Pickering emulsions was realized by forming a thick layer where colloid particles covered the surface of oil droplets. This defending barrier effectively shielded β-carotene from degradation and oxidation. When the CNF concentration of the original suspensions was higher than 2.0 wt.% used in the US-PE samples and 1.6 wt.% used in the HPH-PE samples, retention rates of β-carotene in these samples were relatively high at all storage times, which was in accordance with the storage stability of these samples according to Figure 2B and Figure 3B.

## 4. Conclusions

It was demonstrated in our study that β-carotene-loaded CNF-stabilized Pickering emulsions made by HPH, compared with those made by US, achieved higher stability at the same energy density. The stability of β-carotene-loaded CNF-stabilized Pickering emulsions by HPH owed much to the steric stabilization of CNFs-encapsulating-oils networks. This steric stabilization was more homogeneously generated by the continuous impact force of the HPH process than by the discontinuous cavitation effect of the US process. Once the stability of β-carotene-loaded CNF-stabilized Pickering emulsions made by HPH and US was lost due to creaming, the relatively low retention rates of β-carotene in the emulsions appeared. The different emulsion stabilizing mechanisms between HPH and US can guide technicians to choose the appropriate emulsification techniques. Furthermore, although the encapsulation efficiency of β-carotene in HPH-PE samples was a little lower, the retention rate of β-carotenes was significantly higher than in the US-PE samples. The HPH-treated CNF-stabilized Pickering emulsions provided a stable barrier against coalescence of the oil droplets and photodecomposition of β-carotene. According to the advantages and disadvantages of encapsulation efficiency and retention rate in HPH- or US-prepared Pickering emulsion for encapsulating bioactive components, US and HPH technical integration or the combined emulsifying process innovation should be carried out in the future.

## Data Availability

The original contributions presented in this study are included in the article. Further inquiries can be directed to the corresponding author.

## Abbreviations

The following abbreviations are used in this manuscript:

CNFs	Cellulose nanofibrils
PE	Pickering emulsions
HPH	High-pressure homogenization
HPH-PE	High-pressure homogenization prepared Pickering emulsions
US	Ultrasound
US-PE	Ultrasound-prepared Pickering emulsions
